# Conserved Genes Preferentially Duplicated in Evolution

**DOI:** 10.1371/journal.pbio.0020084

**Published:** 2004-03-16

Over the course of evolution, some organisms have gained many genes and become increasingly complex whereas other, simpler, organisms have survived with comparatively fewer genes. (Compare, for example, the 30,000 genes of humans to the 5,500 of brewer's yeast.) But where do these “new” genes come from? Evolutionary biologists have long known that duplication of existing genes is an important source of genetic novelty—it is easier to copy and modify an existing gene than to create a completely new one from scratch.

Because gene duplication makes such a major contribution to evolution, researchers have attempted to understand the mechanisms of gene duplication, how genes evolve once they become duplicated, and what functional effect gene duplications have for the organism. Recent genomic studies, for example, appear to show that most duplicated genes go through a period of accelerated evolution and also that the presence of duplicated genes adds robustness to the functioning of genomes. In research published in this issue, however, Jerel Davis and Dmitri Petrov look at gene duplication from a different perspective. Rather than asking how genes are duplicated, they asked which genes tend to be “good” at duplicating over the course of evolution. The answer is important for our understanding of the forces underlying gene duplication and will also help us understand why genomes contain duplicates of some genes and not others.

The authors began by identifying duplicated and nonduplicated gene pairs in the yeast Saccharomyces cerevisiae and the worm Caenorhabditis elegans, two model organisms whose genomes have been sequenced. They then looked for the corresponding genes in two distantly related species, the fruitfly and the mosquito, in order to obtain an independent measure of evolutionary rate. This independent measure is vital because of the likelihood that gene duplication itself influences the rate of evolution. After obtaining these rates, the researchers compared the evolutionary rates of duplicated and nonduplicated genes.

Stated simply, the authors found that slowly evolving (that is, more conserved) genes are more successful at generating duplicates than faster evolving genes. This is no recent trend—more conserved genes have been better at generating duplicates of themselves consistently over hundreds of million of years.[Fig pbio-0020084-g001]

**Figure pbio-0020084-g001:**
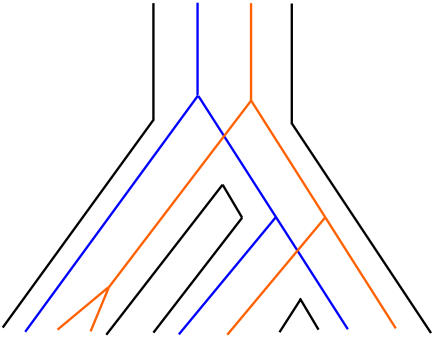
Phylogenetic studies show that slowly evolving genes are more likely to be duplicated than faster evolving genes

This research challenges the assumption that genes are duplicated in an unbiased manner. In addition, it provides the essential background for other genomic studies of gene duplication. For example, the acceleration of protein evolution upon duplication is likely to be even more dramatic considering that it is the slowly evolving genes that duplicate preferentially.

These findings also open up new questions in the study of gene duplication. The authors convincingly demonstrate the bias toward conserved genes in the process of duplication, but how and why does this happen? For a duplicated gene to be retained in a species, the duplicate must be fixed in the population and then must be preserved by natural selection. The preferential duplication of slowly evolving genes might come from a bias in either of these steps, and the authors outline several models for why this might be the case. Further analysis may enable researchers to test these and other models for gene duplication—especially as more sequence data become available—and learn more about this potent phenomenon in genome evolution.

